# Which Patients with COPD Would Benefit from Cough Monitoring?

**DOI:** 10.3390/jcm14134506

**Published:** 2025-06-25

**Authors:** Albertus C. den Brinker, Susannah Thackray-Nocera, Michael G. Crooks, Alyn H. Morice

**Affiliations:** 1 Independent Researcher, NL-5708 DJ Helmond, The Netherlands; 2 Centre for Clinical Science, Hull York Medical School, University of Hull, Cottingham HU16 5JQ, UK; susannah.thackray-nocera@nhs.net (S.T.-N.); michael.crooks@nhs.net (M.G.C.); a.h.morice@hull.ac.uk (A.H.M.)

**Keywords:** COPD, exacerbation, alert, cough, patient data, logistic regression, stratification

## Abstract

**Background:** Cough monitoring for exacerbation detection is optimally effective if used for the appropriate cohort of chronic obstructive pulmonary disease (COPD) patients, i.e., increased cough during exacerbation and prodrome is a prerequisite enabling (early) detection. **Methods:** A post hoc analysis of data from a validation study on an alert system for exacerbation detection based on nighttime cough was used to study if patient data were predictive for the increased cough during exacerbation and for cough count distribution. The quantitative effect on the performance of the alert system when using patient stratification was studied as well. **Results:** Patient data were not predictive for robust cough statistics: neither the nighttime cough count median nor the interquartile range were found to have statistically relevant correlation with the available patient data. Patients with and without increased cough during exacerbation did show differences in their characteristics. Using patient age and symptom questionnaire data, a classifier based on a logistic regression model was parametrised having an accuracy of 85% in predicting presence or absence of increased cough during exacerbation. Using the classifier for patient stratification, the performance of the exacerbation alert system increased with sensitivity going from 59 to 76%. **Conclusions:** The post hoc analysis suggests that patient data can be used to stratify COPD patients for cough monitoring.

## 1. Introduction

Improvements in treatment and management of chronic obstructive pulmonary disease (COPD) may come from various areas [1], such as advanced materials [2] and effective policies [3]. Here, we consider the option of improved monitoring of patients by an unobtrusive exacerbation alert system on the basis of nighttime cough counts. The alert system can be used by caregivers, but may also serve as a tool for improved self-management as patients have difficulties in recognising early symptoms of deterioration [4,5,6].

Coughing is a symptom of many respiratory diseases and automated cough monitoring may be an element in a patient management system. A recent overview paper on cough monitoring [7] stated that assessment of cough is critical in the evaluation of people with chronic respiratory diseases as well as to evaluate the effectiveness of interventions. The paper characterised the current state of the art as in need for further evaluation of objective cough monitoring systems for research and clinic application. In the meantime, the alert system for COPD exacerbation proposed in [8] has been validated. The results [9] showcase the first unobtrusive monitoring system with alerts raised solely on the basis of nighttime cough counts. The system distinguishes itself from other alert systems [10,11,12,13] by its truly passive nature.

To create alerts, the developed system for acute exacerbation of COPD (AE-COPD) uses a time series of cough counts as the only data source. The system proved successful in identifying the majority of exacerbations, yet missed some exacerbations mainly because some patients did not show an elevated cough count during exacerbations. For 44% of the detected exacerbations, the system gave an early alert with a lead time of 4 days or more.

The aim of this paper is to relate cough characteristics to patient data. The notion here is that supplying users with an AE-COPD alert system would preferably be supported by defining expectations of its effectiveness. The reported alert system proved most effective for patients having a relatively high cough count [9] and for those having an elevated cough count in response to an (impending) exacerbation.

An elegant solution would be to screen COPD patients such that we provide cough systems only to patients that benefit most from cough monitoring. We therefore looked into all patient data available at the start of the monitoring period to see if these provide clues to the expected nighttime cough count distribution and presence of increased cough during exacerbation. The patient cohort was split into patients where elevated cough count accompanied an exacerbation and those with a steady cough count. Predictability of this would imply a means of determining upfront which patients to address and withholding devices from patients where no or little measurable effect is expected.

## 2. Methods and Materials

The data used in this post hoc analysis are those from the trial described in [9] and this paper addresses the secondary aim of the trial: improvement of the alert mechanism. The study was reviewed and approved by the Internal Committee Biomedical Experiments of Philips Research and the North East -York Research Ethics Committee, the United Kingdom Health Research Authority (REC Ref.: 21/YH/0203). Informed consent was obtained from all participants involved in the study. Details of the trial set-up are covered in [9]. The trial was a double-blind prospective longitudinal study of continual cough monitoring in COPD patients with a primary aim to validate an alert mechanism for exacerbations based on cough trend data. The study duration was 12 weeks and if no exacerbation occurred in this period, participants were asked to continue for a further 12 weeks. Patients had a clinical diagnosis of COPD using GOLD criteria, two or more moderate and/or severe exacerbations of COPD in the previous year, and a smoking history of 10 packyears or more. The principle investigator decided on the subject’s ability to comply with trial procedures considering significant comorbid medical or psychological conditions. A research nurse checked device operation, collected questionnaire data, and filled out the incident report forms during monthly visits. At the end of the data collection stage, 32 datasets were available for cough count analysis.

**Figure 1:**
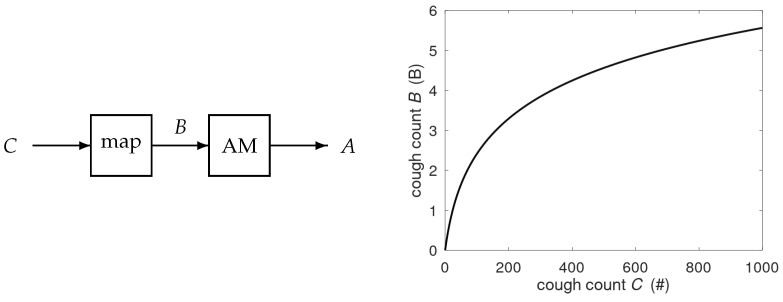
AE-COPD alert system. The nighttime cough counts *C* are mapped to the B-scale and processed by the alert mechanism (AM) to produce alerts *A*. The mapping C→B is shown in the righthand plot, where # indicates the number of coughs observed in a session.

The cough monitor system is passive, i.e., requiring no input from the patient and installed as a stationary device in the patient’s sleeping quarters. Nighttime cough counts were extracted in sessions running from 9 PM to 9 AM. The total amount of coughs in a session is called *C* and is obtained using a personalised cough classifier. The cough counts were mapped to the B-scale, a logarithmic transformation according to

(1) $$B=\alpha \log_{10}\left\{1+\beta C\right\},$$

with α=3.45 and β=0.04. The B-scale is preferred as it allows for patient-independent threshold settings in the alert mechanism [8]. The time series *B* is input to the alert mechanism creating alerts *A*. For cough counts *C* up to 1000, the corresponding *B* stays below 6B, see Figure 1. The alert mechanism smoothes the time series and creates a baseline based on past data. An alert is raised if the smoothed cough count exceeds the time-dependent baseline by a fixed offset twice in three consecutive days.

**Figure 2:**
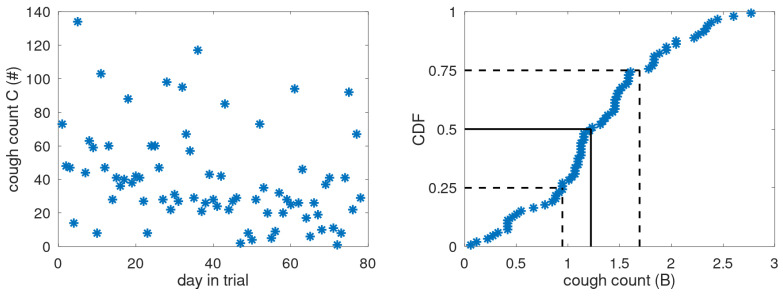
Example of cough counts *C* and the cumulative distribution of the mapped experimental data *B* (blue asterisks) with indications of median (solid black line) and quartiles (dashed black lines).

Even if the patient’s respiratory condition is stable, cough counts are highly variable and best characterised by their distribution. To describe the cough distribution from experimental data, we use the two prime metrics that define a distribution: the average and the spread, where, in view of the high day-to-day variability and potential periods of deteriorated health, we use robust statistics: the median and the interquartile range (IQR). These metrics are presumed to represent the steady-state cough behaviour. An example of cough data is shown in Figure 2 where the cough count *C* is shown as a function of trial day. A large day-to-day variation is observed in line with other studies [8,14]. The cumulative distribution function (CDF) of *B* is shown on the righthand side including position of the median (solid black line) and interquartile range (between the dashed lines).

The correlation between the cough count statistics and available patient data was studied. Reported are the correlation coefficients and their statistical significance (*p*-value) to test the $H_{0}$ hypothesis: absence of correlation between cough statistics and patient data.

Two subgroups were created with patients having increased cough or not during exacerbation. For the two considered cohorts, statistics of patient data were compared in the form of boxplots and a logistic regression model was parametrised. The parametrisation works as follows. The two groups are provided with a binary groundtruth label: E-IC or E-SC, denoting increased or stable cough during exacerbation, respectively. A linear regressor is created as

(2) $$Q=\beta_{0}+\sum_{i=1}^{I}\beta_{i}F_{i},$$

where $F_{i}$ is a feature from the patient data (e.g., age or height), *i* denotes an index associated to a certain feature (i=1,2,…,I), and $\beta_{i}$ are the model parameters (i=0,1,…,I). A logistic function converts the linear combination *Q* to a probability called *p*, (0≤p≤1). Given a set of patient data covering both groups, the parameters are optimised using the maximum likelihood criterion.

Setting a threshold on *p*, a classifier is created. We take threshold T=0.5, generating binary output label *Y* according to

(3) $$Y=\left\{\begin{array}{cc} E\text{-}IC, & \mathrm{if}\;p\leq T,\\ E\text{-}SC, & \mathrm{if}\;p>T. \end{array}\right.$$

In this way, the logistic regression model provides a binary label to each patient based on their features. A confusion matrix is used to report the performance of this classifier: it counts the number of patients for each of the four possible combinations of groundtruth and estimated binary label. From the confusion matrix, classification performance metrics like sensitivity, specificity, accuracy, etc., can be generated.

To assess the effect of deployment of the developed classifier, all 32 patients were stratified. The performance of the alert mechanism was recalculated using the stratified cohort. For completeness, we recap the methods used to identify exacerbations and how the association between alerts and exacerbations was made [9].

Exacerbation identification was patient-initiated with the start date of a moderate AE-COPD defined as the date that the participant reports starting steroids and/or antibiotics for their chest or the date that a prescription for steroids and/or antibiotics is issued, excluding renewal of ‘just-in-case’ medications. The date that the participant reports taking their last dose of steroids and/or antibiotics is the end date. The start and end-dates for severe AE-COPD are defined by the start and end dates of treatment (as per moderate AE-COPD) or the duration of hospitalisation (whichever is longer).

The exacerbations are treated as singular events and not as a series of (independent) exacerbation days. Alerts are also considered as alert trains rather than independent events each day. The reasons to do so and its consequences are the following. Working on a day-basis would require creating a groundtruth for each single day, but defining the groundtruth for prodrome and convalescence makes this option unattractive. Furthermore, a single alert for an (upcoming) AE-COPD would suffice; there is no need that the alerts last as long as the defined exacerbation period.

**Figure 3:**
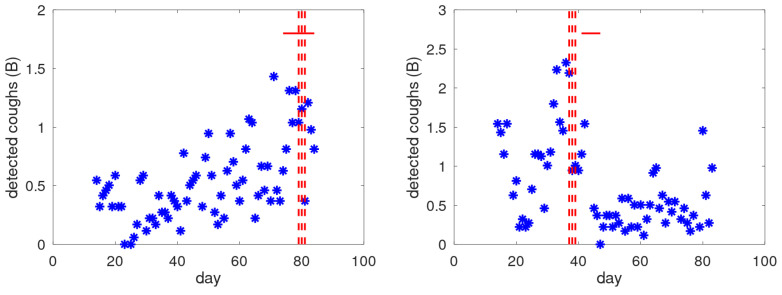
Two examples of cough counts, exacerbation period and alerts. Cough data are presented on the B-scale (i.e., the input to the alarm mechanism); each asterisk presents the total cough count over a nighttime session. The solid horizontal red line indicates the exacerbation period. The dashed vertical red lines indicate the days an alert is raised. (**Left**) example of a late alert: the alerts are raised during the exacerbation period. (**Right**) example of an early alert: the alerts precede the exacerbation period.

The way that exacerbation events and alert trains were coupled is the following. If an alert train starts in the fortnight of the exacerbation onset, this is called an early alert. If an alert train starts during the exacerbation period, it is called a late alert. Early and late alerts together define the correct detections. Exacerbations without early or late alert are counted as missed alerts. All other alert trains are defined as false alerts. To prevent investigator bias, the study was designed as a double-blind study: only cough monitor data but no medical data were available for alert train generation and no cough monitor data (raw or analysed) were available when defining exacerbation periods. Two examples of cough counts (on the B-scale, i.e., input to the alert mechanism) are provided in Figure 3, together with indication of alert days and exacerbation period. Note that for the first 13 days, no cough counts are provided. The alert mechanism requires this period to initialise its baseline definition. The left graph indicates a late alert: the alert days fall within the exacerbation period. In the right graph, the alerts precede the exacerbation period.

The alert mechanism is, therefore, an event-based, one-class classifier with default outcome ‘no exacerbation’. For a one-class classifier, the performance metrics sensitivity and positive predictive value (PPV) remain valid. However, since there is no second class, the concept of specificity (and accuracy) does not exist and is replaced by the number of false alerts. As alerts are raised along the time axis, it is convenient to translate the number of raised alerts into a frequency of false alerts by dividing the number of false alerts by the observation period. The PPV and false alert rate are considered as metrics of prime importance. Trust in the system and patient adherence are likely degraded for a low PPV or a high false alert rate.

## 3. Results

**Figure 4:**
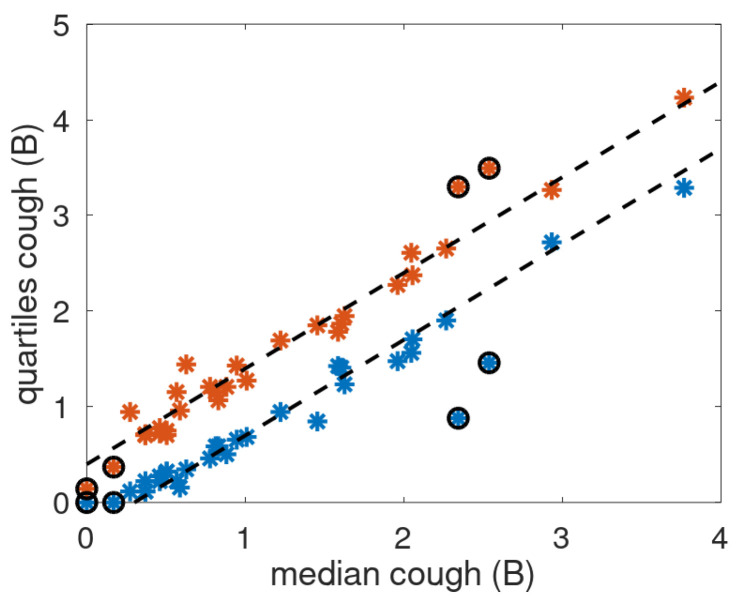
Cough quartiles as a function of median cough count. Blue and red blue asterisks: first and third quartile, respectively. Dashed lines: median plus offset of −0.4 and 0.35 B. Black circled asterisk: data excluded from correlation analysis.

The considered nighttime cough statistics consist of the median and interquartile range for the 32 patients with proper cough data *B*. The cough statistics are shown in Figure 4. The quartiles follow the straight dashed lines with some exceptions. There are two data points with very large IQR and two data points with first quartile equal to zero, a very low median value, and small IQR. These four cases were considered outliers and excluded from the correlation analysis.

The patient data (Table 1) consisted of Age, Height, Weight, BMI, CAT, Packyears, VAS, HARQ, FEV1 (base and %), and admission and exacerbation frequencies. Correlation coefficients and the associated *p*-value of these data with nighttime cough median and IQR were calculated. The correlation values are low, and the range of *p*-values for the median cough was 0.07–0.98 and for the IQR 0.13–0.98. All *p*-values are above 0.05, meaning no significant correlation was found.

The patient data were subdivided into three subsets. Patients with an exacerbation during cough monitoring were selected, setting the patients without exacerbation aside. The group with exacerbation was split according to increasing cough count or not before or during the exacerbation. Identification of increased cough was performed partly based on alerts raised by the alert mechanism as this implies increased cough. Also visual inspection was performed as part of the cough monitoring period was not covered by the alert mechanism due to delay caused by baseline creation. Two patients with a low median and interquartile cough count (see Figure 4) were excluded as the presumed cause is absence of the patient in the indicated sleeping quarters. There were 13 patients with increased cough (E-IC) and 7 patients with steady cough (E-SC) trends during the exacerbation periods.

**Figure 5:**
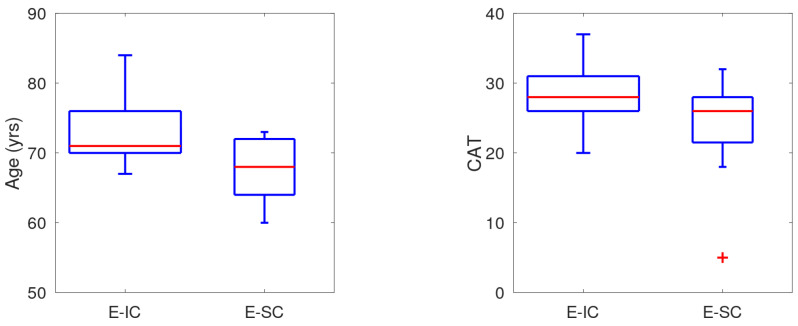
Boxplots of Age and CAT score over the E-IC and E-SC subgroups. E-IC: patients with increased cough during exacerbation; E-SC: patients with stable cough during exacerbation. The + symbol identifies an outlier in the observed data.

Patient statistics of these subsets were compared in a boxplot and shown for Age and CAT score in Figure 5. For these patient data, the medians are markedly different though the distributions overlap partly: higher age and CAT score are associated with increased coughing during exacerbation. With Age and CAT score, a logistic regression model was parametrised and the prediction results of a classifier using threshold setting T=0.5 are given by the confusion matrix in Table 2. For 17 out of the 20 patients, the correct prediction was made, meaning an accuracy of this stratification of 85% and the PPV was 86% (12 out of 14).

To quantify the effect of using the stratification on the performance of the alert mechanism, we applied the designed classifier on all 32 patients and compared its results with the earlier reported performance metrics [9]. We counted the number of days the alert mechanism was operating (e.g., excluding initial delay due to baseline estimation), the exacerbations occurring in this period, the number of detected exacerbations (split into late and early), the number of missed exacerbations, and the additional alerts (see Table 3). The stratification reduces the number of patients, the number of monitored days, and the number of exacerbations. The screening did not affect the number of detections, but almost halved the number of misses. For the false alerts, we provide a range instead of a number as alert trains ran consistently over a number of consecutive days, which may indicate a medical issue that has gone unobserved [9]. Overall, there is a small increase in the false alarm rate: while for the original cohort, one false alert occurred on average every 12–22 months, it became 10–20 months for the screened cohort.

## 4. Discussion

### 4.1. Correlation Analysis

Four patients were excluded from the correlation analysis. The two patients with large IQR were patients having multiple exacerbation in the monitoring period with one of them also having a relatively short monitoring period. Even robust statistics are unlikely, therefore, to reflect the normal respiratory condition. For the two patients with very low median cough count, there are many days without observed coughs and a very quiet acoustic environment. This may indicate that these patients were not always occupying their sleeping quarters.

None of the patient data had a significant correlation to the objective nighttime cough counts. Correlations with the cough statistics on the B-scale were presented, and similar results hold for the cough counts *C*. Even the cough VAS, a priori the most likely candidate as it is a direct reflection of cough severity, does not reveal any correlation to the cough count statistics. It shows that objective cough count is an independent source of information on respiratory health status. For our monitor, the lack of correlation is unfortunate as upfront information on the average nighttime cough count gives information on the success of providing early alerts [9].

### 4.2. Increased Cough During Excerbation

The boxplots of Age and CAT score demonstrate the disparity of the distributions for the considered groups of patients with and without increased cough during exacerbation. Caution is needed as the data were limited. The logistic regression classifier designed using Age and CAT score performs well, having an accuracy of 85% and PPV of 86%. The regressor uses three parameters only, making the risk of overfitting minimal. The patient classifier is a valuable asset to cough monitoring as it enables targeted monitoring by screening of patients. Without screening, the alert system does not indicate the presence of an exacerbation for 30% of the cases due to absence of increased cough. A logistic regression model with VAS instead of CAT score was also successful but VAS is a much less common measurement. Validation of our findings is recommended in view of the limited cohort size.

### 4.3. Patient Stratification

The comparison of the alert performance over the full cohort (including patients excluded from the training of the patient classifier) with that after stratification confirms the effectiveness of the screening. Even though the stratification is imperfect with an accuracy of 0.85, the fraction of detected exacerbations increased from 59 to 76% without a substantial increase in the false alarm rate. Stratification also implies preventing a healthcare burden because fewer devices need to be installed and maintained.

## 5. Conclusions

We considered the relation between patient data and nighttime cough characteristics. Correlations between statistics of nighttime cough and patient data were determined but no significant correlation was observed. Even for the most likely candidate, the cough VAS, no significant correlation was found. This underpins that objective cough count is an independent source of information.

Though the patient data were not predictive for the nighttime cough count distribution, it was found to be predictive for whether patients have increased cough during exacerbation. Age plays an important role here as well as subjective data (CAT score). A classifier based on logistic regression of these patient data gave 85% accuracy. Patient stratification enables reduction in installed devices and boosts the performance of the COPD exacerbation alert system. In view of the small sample size, validation of the findings is recommended.

## Figures and Tables

**Table 1:** Correlation coefficient and *p*-value between cough statistics (nighttime median and interquartile range (IQR)) and various patient data. Packyears: lifetime tobacco exposure; CAT: COPD Assessment Test; HARQ: Hull Airway Reflux Questionnaire; VAS: Visual Analogue Scale for cough severity; FEV1: Forced Expiratory Volume in 1 s. Exacerbation and admission refer to number of occurrences over the last 12 months. FEV data were available for part of the cohort (*n* = 13).

		Cough Median		Cough IQR	
	n	Correlation	*p*-Value	Correlation	*p*-Value
Age	28	0.16	0.40	0.00	0.98
Weight	28	−0.34	0.08	0.18	0.35
Height	28	−0.35	0.07	−0.11	0.56
BMI	28	−0.19	0.32	0.29	0.13
Packyears	28	−0.21	0.28	0.00	1.00
CAT	28	−0.07	0.72	−0.18	0.36
HARQ	28	0.00	0.98	0.05	0.80
VAS	28	0.09	0.66	0.11	0.58
Exacerbation	28	−0.03	0.89	0.22	0.25
Admission	28	0.16	0.43	0.00	0.99
FEV1 base	13	0.13	0.68	0.24	0.42
FEV1 %	13	0.22	0.47	0.27	0.38

**Table 2:** Patients with and without increased cough during exacerbation: observed versus classification based on patient characteristics. Confusion matrix for classifier using logistic regressors Age and CAT score with threshold setting T=0.5. The 2×2 matrix gives the number of patients for each of the four possible combinations of groundtruth and predicted label. E-IC: patients with increased cough during exacerbation; E-SC: patients with stable cough during exacerbation.

			Predicted	
			E-IC	E-SC
Groundtruth	E-IC	13	12	1
	E-SC	7	2	5

**Table 3:** Performance numbers over original and screened cohort. Late and early alerts represent the subdivision of detected alerts into alerts before and during the patient-initiated identified exacerbations.

Patients	Days	Exacerbations	Alerts				
			Missed	Detected	Early	Late	False
32	2694	27	11	16	7	9	4–7
22	1817	22	6	16	7	9	3–6

## Data Availability

The data presented in the study are stored securely at Hull York Medical School. The investigators act as custodians for the data processed and generated by the study and they are also responsible for access to any information included. Requests can be made to A.H. Morice. Due to privacy and institutional regulations, the data are not publicly accessible.

## Abbreviations

The following abbreviations are used in this manuscript:

AE-COPD	Acute Exacerbation of COPD
CDF	Cumulative Distribution Function
COPD	Chronic Obstructive Pulmonary Disease
CAT	COPD Assessment Test
E-IC	Patient Subset having Exacerbations with Observed Increased Cough
E-SC	Patient Subset having Exacerbations with Observed Steady Cough
FEV1	Forced Expiratory Flow in 1 s
HARQ	Hull Airway Reflux Questionnaire
IQR	Interquartile Range
PPV	Positive Predictive Value
VAS	Visual Analogue Scale
